# The Treatment of Incomplete Abortion

**Published:** 1888-03

**Authors:** A. E. Aust Lawrence

**Affiliations:** Physician-Accoucheur to the Bristol General Hospital, and Lecturer on Diseases of Women, Bristol Medical School


### THE TREATMENT OF INCOMPLETE ABORTION.

BY

A. E. Aust Lawrence, M.D.,

*Physician-Accoucheur to the Bristol General Hospital, and Lecturer on Diseases of Women, Bristol Medical School.*

---

Having had a rather large experience in the treatment of these cases, and feeling that the profession, as a rule, have not very definite ideas on the subject, I am induced to place before my readers, in a very brief and practical manner, a line of treatment which has proved of service in my own practice. In the first place, I shall assume that the question of the patient being pregnant is set at rest in the affirmative; or, at all events, she has missed one period at least, and is now suffering from uterine hæmorrhage, continuous and possibly excessive. If there is one thing more than another I am certain about, it is this, that no woman ought to be allowed to go on bleeding in an irregular manner without a vaginal examination being made; and if no cause for the bleeding be found *outside* the uterus, then the *inside* of that organ should be examined.

I have heard medical men state that they have never seen any ill results occur from leaving their miscarriage cases to Nature. All I can say is, that I have seen women almost die from the *primary* hæmorrhage of an abortion, and many broken down completely from continuous hæmorrhage, and not a few have died from septicæmia. I regard the treatment of an abortion case in as serious a light as any confinement at full term, and yet many a man who would manage the latter makes a sad bungle over the former. I consider the treatment laid down in books, as a rule, to be highly misleading; the authors are too vague in their directions, and sometimes, to my mind, give very ill-advised directions for treatment, especially when they tell practitioners to plug the vagina, &c., which I consider is very bad practice, unless you cannot possibly plug the cervix uteri.

The treatment I lay down is very simple.

We will assume that the case has gone beyond the expectant line of treatment—that, in spite of rest, &c., the bleeding continues. This is what should be done. Make the incomplete abortion complete by clearing out the uterus; and if this is done in the way I advise, the results will in all cases be satisfactory. Put the patient on her left side (Sims's position), wash out the vagina with carbolic lotion 1 in 100, pass a duck-bill speculum, take hold of the anterior lip of the cervix with Sims's hook, and pass up into the uterus a bougie of 20 grains of iodoform; then one of my antiseptic and gelatine-coated sponge tents—retain this in the cervix uteri by a piece of iodoform wool in the vagina. In from twelve to twenty-four hours see the patient again. Remove the vaginal plug and the sponge-tent, both of which will be perfectly aseptic; wash out the vagina with the carbolic lotion, and now pass the finger into the uterus, and very likely you will be able to reach the fundus and clear out the contents. After this, wash out the uterus with carbolic lotion, and give directions for the vagina to be daily syringed with the same lotion. What I have described can be done in most cases. I

will allude later on to some difficulties, and how to meet them. The great secret in the treatment is to clear out the uterus. I have just stated how I consider it ought to be done. I will now state how I consider it ought *not* to be done; and I only do so as I find it is often practised to the great detriment of the patient, and the deaths arising from the abuse of clearing out the uterus determine some men to let their patients die by Nature rather than by Art. I take every antiseptic precaution; the treatment I condemn takes none at all. A tent (not an antiseptic one, like mine) is pushed up the vagina (which has not been washed out) with one or other of the various tent-holders (most useless kind of things), and carried into the uterus with the decomposing discharges of the vagina. This septic tent is plugged in by an ordinary piece of sponge, or common non-antiseptic wool; the next day the plug and tent are removed, smelling most horribly, and the finger is at *once* pushed up into the uterus and the cavity cleared of its contents, possibly aided by some of those wretched and dangerous instruments called ovum forceps. The uterus is not washed out, and the patient dies of septicæmia in a few days, or has a lingering convalescence after pelvic abscess, &c. This is not a fancy picture. I have re-repeatedly seen these results both in hospital and private practice; and because some men, either through ignorance or wilfully, carry out this line of treatment, and their patients die, it is no justification for letting women drift into danger, because, with the precautions I have named, the treatment I advocate is both safe and sure. The two great things to bear in mind are—gentleness in manipulation (fingers before forceps), and complete antiseptic precautions. I could put on record any number of cases to illustrate this paper, but I consider the *Journal*

is too valuable to be padded with material not required. The remarks I have made are founded on experience from actual cases. There are a few points of practical interest in the management of some cases which may be of service, so I will mention them. First: In some cases it is impossible to clear the uterus from all fragments of ovum, &c.; in these cases, keep the inside of the uterus aseptic by passing daily a piece of iodoform bougie, and in a few days the material will be thrown off perfectly sweet. These are the cases that die of septicæmia, if care is not taken, because the remains of the ovum decompose *in utero*, and sepsis arises. As a rule, if these precautions are adopted, no trouble ensues.

Sometimes it is almost impossible to pass the finger up to the fundus to clear out the uterus; in these cases, a good plan is to partly retrovert the uterus, and then, with the fundus pressed against the sacrum, it is possible to get the finger right up, and clear out the uterine cavity. I prefer this way to pulling down the uterus with vulsella. The only instrument I use inside the uterus in these cases is a blunt curette, and I very seldom use that. Very often, in your manipulations to get out a piece of the ovum, you will find great assistance by passing along the side of your finger which is in the uterus a catheter, and injecting hot water. This stimulates the uterus to throw off its contents; and I have frequently found that the os uteri dilates at the same time, enabling you to get in two fingers, whereas before you could only get in one. In conclusion, I would urge all those who have the conduct of a case of abortion to give this treatment, with its precautions, a fair trial, and I am sure it will be to the benefit of the patient.

